# Increased basal ganglia binding of ^18^ F‐AV‐1451 in patients with progressive supranuclear palsy

**DOI:** 10.1002/mds.26813

**Published:** 2016-10-06

**Authors:** Ruben Smith, Martin Schain, Christer Nilsson, Olof Strandberg, Tomas Olsson, Douglas Hägerström, Jonas Jögi, Edilio Borroni, Michael Schöll, Michael Honer, Oskar Hansson

**Affiliations:** ^1^ Skåne University Hospital, Department of Neurology Lund Sweden; ^2^ Lund University, Clinical Memory Research Unit, Department of Clinical Sciences Malmö Malmö Sweden; ^3^ Skåne University Hospital, Department of Radiation Physics Lund Sweden; ^4^ Skåne University Hospital, Department of Clinical Neurophysiology Lund Sweden; ^5^ Skåne University Hospital, Department of Clinical Physiology and Nuclear Medicine Lund Sweden; ^6^ Roche Pharmaceutical Research and Early Development, Neuroscience Discovery & Biomarkers, Roche Innovation Center Basel Switzerland; ^7^ MedTech West and the University of Gothenburg, Department of Clinical Neuroscience Gothenburg Sweden; ^8^ Skåne University Hospital, Memory Clinic Malmö Sweden

**Keywords:** Progressive supranuclear palsy, tau, positron emission tomography, basal ganglia

## Abstract

**Background:**

Progressive supranuclear palsy (PSP) is difficult to diagnose accurately. The recently developed tau PET tracers may improve the diagnostic work‐up of PSP.

**Methods:**

Regional tau accumulation was studied using ^18^F‐AV‐1451 PET in 11 patients with PSP and 11 age‐matched healthy controls in the Swedish BioFinder study.

**Results:**

^18^F‐AV‐1451 standard uptake volume ratios were significantly higher in the basal ganglia in PSP patients when compared with controls (globus pallidus 1.75 vs 1.50; putamen 1.51 vs 1.35). Retention in the basal ganglia was correlated with age in both groups (*r* = .43–.78, *P* < .05). In PSP, we observed a significant correlation between clinical deterioration measured with the PSP rating scale and standard uptake volume ratios in the globus pallidus (*r* = .74, *P* < .05). However, no ^18^F‐AV‐1451 retention was observed in the cerebral cortex or white matter of either PSP patients or controls, and autoradiography did not reveal any specific binding of AV‐1451 to PSP tau aggregates.

**Conclusion:**

We found higher ^18^F‐AV‐1451 retention in the basal ganglia of PSP patients when compared with healthy elderly controls, but also increases with age in both controls and patients. As a result of the overlap in retention between diagnostic groups and the age‐dependent increase present also in controls, ^18^F‐AV‐1451 PET might not reliably distinguish individual patients with PSP from controls. However, further studies are needed to evaluate whether ^18^F‐AV‐1451 PET might be useful as a progression marker in clinical PSP trials. © The Authors. Movement Disorders published by Wiley Periodicals, Inc. on behalf of International Parkinson and Movement Disorder Society.

Progressive supranuclear palsy (PSP) is a rare neurological disorder resulting in rigidity, bradykinesia, bradyphrenia, recurrent falls, and oculomotor abnormalities.^1^, ^2^ The neuropathology is characterized by tau‐positive inclusions containing primarily the 4‐repeat (4R) isoform of tau and regional brain atrophy affecting the basal ganglia, the frontal lobe, and the dentate nucleus.^3^, ^4^ During the early stages of disease progression, it is often difficult to distinguish PSP from Parkinson's disease, multiple system atrophy, or corticobasal degeneration, and consequently a significant proportion of patients with PSP does not receive a correct diagnosis.^5^ Cerebrospinal fluid levels of neurofilament might distinguish atypical parkinsonism from Parkinson's disease but do not discriminate between PSP, multiple system atrophy, or corticobasal degeneration.^6^ Methods using advanced magnetic resonance imaging (MRI) to diagnose atypical parkinsonian disorders are being developed but have not yet been fully established.^7^ Positron emission tomography (PET) ligands binding to tau aggregates might prove useful for improving the diagnosis of different tauopathies, including PSP. In recent years, the advent of the tau tracers ^18^F‐AV‐1451 (T‐807),^8^ ^18^F‐THK‐5351,^9^ and (2‐((1*E*,3*E*)‐4‐(6‐(11C‐methylamino)pyridin‐3‐yl)buta‐1,3‐dienyl)benzo[*d*]thiazol‐6‐ol) (^11^C‐PBB3^10^) has increased the interest in tau PET imaging in neurodegenerative diseases, but their clinical utility is not fully known to date.

To assess the *in vivo* binding of ^18^F‐AV‐1451, we performed PET examinations in 11 patients with PSP and 11 age‐matched controls. Furthermore, using autoradiography, we assessed ^3^H‐AV‐1451 binding to tau aggregates in tissue from the frontal cortex of PSP patients and healthy controls and from the putamen of a patient with PSP.

## Methods

### Participants

Patients were recruited from the Neurology Clinic and the Memory Clinic at Skåne University Hospital, Malmö, and Lund, Sweden. Informed written consent was obtained from all patients before inclusion in the study. All procedures conformed to the Declaration of Helsinki and were approved by the ethics committee at Lund University as well as the Swedish Medical Products Agency. A total of 11 patients with PSP were recruited along with 11 neurologically healthy, age‐matched controls. Of the 11 PSP patients, 9 were diagnosed with Richardson's syndrome according to the National Institute of Neurological Disorders and Stroke criteria,^11^ 2 of the patients were diagnosed with PSP‐parkinsonism.^2^ Controls were recruited from the Swedish Biofinder study (http://www.biofinder.se). Amyloid status was assessed using either lumbar puncture or 18F‐Flutemetamol PET (see supplementary table for details). The patients were assessed with Hoehn and Yahr staging, Schwab and England activities of daily living, and PSP rating scales as well as by detailed neurologic and psychiatric exams conducted by a physician experienced in movement disorders. The study participants' cognitive functions were assessed by a registered research nurse using cognitive tests including the Mini Mental State Examination. Clinical data are summarized in Table 1.

**Table 1:** Clinical data and regional SUVRs

	Healthy controls, n = 11	PSP, n = 11
Age, y	70.9 ± 1.9	70.7 ± 2.2
Disease duration, y	–	5.27 ± 2.6
MMSE, median (range)	30 (27‐30)	29 (22‐30)^a^
Hoehn & Yahr, median (range)	–	4 (2‐5)
Schwab & England, median (range)	–	50% (30‐80)
PSPRS, median (range)	–	40 (24‐64)
Midbrain volume, cm^3^	6.40 ± 0.23	4.77 ± 0.24***
Pons volume, cm^3^	14.34 ± 0.72	13.27 ± 0.68
Midbrain/Pons ratio	0.45 ± 0.02	0.36 ± 0.01***
SUVR, mean ± SEM		
Caudate	1.10 ± 0.04	1.22 ± 0.03*
Putamen	1.35 ± 0.05	1.51 ± 0.04*
Globus pallidus	1.50 ± 0.06	1.75 ± 0.05**
Thalamus	1.12 ± 0.02	1.22 ± 0.02**
Frontal lobe	1.00 ± 0.02	0.98 ± 0.01
Occipital lobe	1.06 ± 0.02	1.07 ± 0.02
Parietal lobe	1.01 ± 0.02	1.01 ± 0.02
Temporal lobe	1.10 ± 0.02	1.07 ± 0.01
Cingulum	1.01 ± 0.01	0.99 ± 0.01
Frontal white matter	1.05 ± 0.05	0.98 ± 0.03
Dentate nucleus	1.21 ± 0.03	1.29 ± 0.05
Midbrain	1.14 ± 0.03	1.25 ± 0.03*
Pons	0.91 ± 0.02	0.91 ± 0.03

MMSE, mini‐mental state exam; PSPRS, PSP rating scale; SEM, standard error of the mean; SUVR, standardized uptake value ratio.

a Two participants in the PSP group could not complete MMSE testing because of aphasia.

*P < .05; **P < .01; ***P < .001.

### Imaging

#### MR Imaging and Volumetry

All patients underwent MRIs on a 3.0T Siemens Skyra scanner (Siemens Medical Solutions, Erlangen, Germany). Acquired sequences were T1‐weighted magnetization‐prepared rapid gradient echo and fluid‐attenuated inversion recovery. Because the midbrain volume is decreased with increasing disease duration in PSP,^12^ midbrain and pons regions of interest (ROIs) were delineated on sagittal T1 magnetization‐prepared rapid gradient echo images in PMOD, version 3.603 (PMOD Technologies LLC, Zurich, Switzerland) and volumes were estimated (see supplementary methods for further details). The MRI voxel‐based morphometry is described in detail in the supplementary methods.

#### PET

The radiosynthesis procedure and radiochemical purity are described in detail in the supplementary methods. For each PET exam, the participant was fixated to the PET system using a dedicated head holder to prevent head motion during the acquisition. A low‐dose computed tomography (CT) scan was performed prior to each PET scan for attenuation correction. The radioligand was then administered intravenously as a bolus injection of 372 ± 14 MBq of ^18^F‐AV‐1451. Emission data were acquired in list‐mode for approximately 3 hours using a GE Discovery 690 PET/CT system (GE Healthcare, Milwaukee, Wisconsin). Acquisition was divided into 3 dynamic scans (0‐60 minutes, 80‐140 minutes, and 160‐180 minutes), with a 20‐minute break at 60 and 140 minutes postinjection. Each of the 3 measurements was preceded by a new transmission scan to account for shift in head position across measurements.

Each dynamic acquisition was temporarily reconstructed without attenuation correction, after which the spatial agreement between the attenuation map and the nonattenuation corrected images was visually inspected. For those cases in which motion could be detected, the PET frame was manually adjusted to fit with the attenuation map, and the corresponding sinogram was adjusted accordingly. Once all acquisitions were considered to be aligned with the attenuation map, the realigned sinograms were reconstructed again, with attenuation correction using an iterative Vue Point HD algorithm with 6 subsets, 18 iterations with 3‐mm filters, and no time‐of‐flight correction. These parameter settings were established using phantom measurements and preliminary analysis of the data. The first of the 3 acquisitions (0‐60 minutes) was reconstructed into the following frame definition sequence: 12 × 10 seconds, 6 × 20 seconds, 6 × 30 seconds, 3 × 60 seconds, 5 × 120 seconds, followed by 300‐second frames until the end of the acquisition. The second (80‐140 minutes) and third (160‐180 minutes) acquisitions were reconstructed into 12 and 4 300‐second frames, respectively.

A total of 7 of the PSP cases and 5 controls underwent a simplified protocol including only 1 dynamic scan for 80 to 120 minutes postinjection.

#### ROI‐Based Analyses of PET Data

PET data was further processed using PMOD version 3.603 (PMOD Technologies LLC, Zurich, Switzerland). PET images were motion corrected using the view tool to account for residual frame‐to‐frame misalignment because of movement. Rigid body affine coregistration of PET and MRI scans as well as MRI gray‐white matter segmentation, spatial normalization, and transformation of template ROIs to PET space were performed using the neuro tool. As a template, the Automatic Anatomic Labelling (AAL)^13^ template was used. Cortical ROIs were masked using a gray matter probability map with a threshold set at 0.75 and transformed to PET space using the affine transformation obtained from MR‐to‐PET coregistration. Because it has been suggested that the AAL template may not be optimal for subcortical structures,^14^ ROIs for the basal ganglia structures, thalamus, dentate nucleus, and frontal white matter were hand‐drawn (see supplement for details) on MRI images and transformed into PET space using the same transformation. Cortical ROIs from the AAL atlas were pooled into frontal, temporal, parietal, occipital, and cerebellar gray matter ROIs (see supplementary methods for details). Cerebellar gray matter, not including the dentate nucleus, was considered a suitable reference region as a result of the absence of tau pathology in this region in PSP patients without ataxia.^15^, ^16^, ^17^ Mean standardized uptake value ratios (SUVRs) were calculated for all patients for the 80‐ to 120‐minute time period.

#### Voxelwise Analysis of PET Data

AV‐1451 uptake patterns were compared between PSP patients and controls using a voxelwise 2‐sample *t* test as implemented in SPM12 (http://www.fil.ion.ucl.ac.uk/spm) using continuous measures of age and sex as covariates. All AV‐1451 SUVR images had been transformed into common Montreal Neurological Institute (MNI) space by using transformation measures from warping the coregistered MRI scans to the 2‐mm MNI152 MRI template (http://www.fmrib.ox.ac.uk/fsl) and smoothed with an 8‐mm full width at half maximum kernel prior to the analysis.

### Statistical Analyses

The SUVR data were found to be normally distributed using the D'Agostino & Pearson omnibus normality test (GraphPad Prism 7, La Jolla, CA, USA). Student's *t* tests were used to assess statistical differences between the groups. Furthermore, an analysis of covariance was used to determine whether the SUVRs of certain regions were still increased in PSP when adjusting for age. Spearman's rank order was used to test correlations in each diagnostic group separately. A linear regression model was used including the whole sample to study associations between regional SUVRs and age when adjusting for diagnosis. Receiver operating characteristic curves were performed to assess the diagnostic accuracy of SUVR in the basal ganglia and midbrain when differentiating PSP patients from controls. *P* values < .05 were considered statistically significant. ROC curves were calculated using SPSS Statistics, version 23 (IBM Corporation, Armonk, NY, USA).

## Results

### Demographics and Volumetric Analyses

There were no differences in age between the PSP patients and controls (Table 1). The average disease duration of the PSP patients was 5.3 years, and they exhibited a median value of 40 (range 24‐64) on the PSP rating scale. As expected, MR imaging revealed significant atrophy of the midbrain in PSP patients (Table 1). Furthermore, a voxel‐based morphometry analysis revealed significant gray matter reductions in PSP relative to controls bilaterally in the frontal medial cortex, frontal pole, anterior cingulum, insular cortex, anterior thalamus, and caudate nucleus (Supplementary Figure 1).

**Figure 1:**
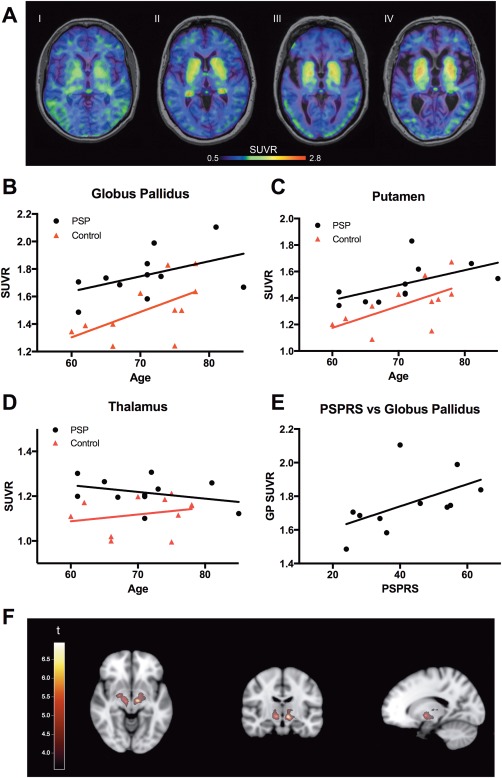
Tau standardized uptake value ratios (SUVRs) and correlation to age. **A**: Averaged ^18^F‐AV‐1451 images over 80 to 120 minutes in 2 control patients (I and II) and 2 PSP patients (III and IV), aged I = 62, II = 70, III = 73, and IV = 81 years. Scale bar denoting SUVR‐values. **B‐D**: SUVRs plotted against age in the globus pallidus (**B**), putamen (**C**), and thalamus (**D**). **E**: Correlation of SUVR values in the globus pallidus plotted against results using the PSP rating scale. **F**: Statistically significant clusters (*P* < .001, uncorrected, k > 50 voxels) resulting from a voxelwise *t* test between AV‐1451 mean images of PSP patients and controls projected on the MNI152 2‐mm brain template. GP, globus pallidus; PSPRS, PSP rating scale. [Color figure can be viewed at http://wileyonlinelibrary.com]

### Comparison of AV‐1451 SUVRs Between Controls and PSP Patients

Using the cerebellar cortex as a reference region, SUVRs were calculated for all participants and regions (Table 1). We found statistically significant differences between PSP patients and healthy controls in the basal ganglia, with the highest SUVR values in the globus pallidus and the putamen (Table 1; Fig. 1A‐C). Moreover, we found significant group differences in the thalamus and the midbrain with slightly higher SUVRs in PSP patients when compared with controls (Table 1, Fig. 1D). The increases in SUVRs in PSP patients in the globus pallidus, putamen, caudate nucleus, thalamus, and midbrain were still significant when adjusting for age (*P* < .05). For both patients and controls, SUVR levels in cortical regions were similar to those observed in cerebellum (Table 1). Receiver operating characteristic curve analysis revealed that AV‐1451 could differentiate PSP patients from controls with area under the curve (AUC) values ranging from 0.74 to 0.84 (globus pallidus, AUC = 0.81; 95% confidence interval [CI] = 0.62‐1.0; putamen, AUC = 0.74 [95% CI = 0.53‐0.96]; caudate, AUC = 0.74 [95% CI = 0.53‐0.96]; thalamus AUC = 0.84 [95% CI = 0.66‐1.0]; midbrain AUC = 0.74 [95% CI = 0.53‐0.96]).

Voxelwise comparisons between PSP patients and controls confirmed our findings in the ROI data, showing significant clusters in the bilateral globus pallidus and thalamus at a significance level of *P* < .001 (uncorrected for multiple comparisons) and a voxel threshold of k > 50 voxels (Fig. 1F).

Furthermore, Supplementary Figure 2 shows transversal slices through the whole brain of the most advanced PSP case (PSP rating scale score 64; diagnosis of PSP confirmed by neuropathology), which revealed clear retention in the basal ganglia only.

### Associations Between AV‐1451 SUVRs and Age

We observed that the signal in the regions with the highest retention of ^18^F‐AV‐1451 was increased in older individuals independent of diagnostic group (Fig. 1A). Consequently, we investigated the correlation between SUVRs and age in the globus pallidus and putamen (Fig. 1B‐C) and found significant positive correlations between the SUVRs and age in both the globus pallidus (*r* = 0.62, *P* < .05) and putamen (*r* = 0.63, *P* < .05) in the control group. In patients with PSP, there was a significant correlation between age and regional SUVR in the putamen only (*r* = 0.78, *P* < .01). In the caudate nucleus, we found a significant correlation between age and SUVRs in the PSP group (*r* = 0.63, *P* < .05), but not in controls (Supplementary Figure 3). When including all participants in a linear regression model and adjusting for diagnostic status, we found significant associations between age and the SUVRs in the globus pallidus, putamen, and caudate nucleus.

### Associations Between AV‐1451 SUVRs and Disease Severity in PSP

Next we studied whether the regional SUVRs were associated with disease severity in PSP. We found a positive correlation between globus pallidus SUVRs and the PSP rating scale values (*r* = 0.74, *P* < .05; Fig. 1E), but not in other regions, including the putamen and thalamus.

### Autoradiography

Autoradiography was performed on the frontal cortex sections of 3 patients with PSP and 3 controls as well as sections from the putamen of 1 PSP patient. We found no specific binding of ^3^H‐AV‐1451 to tau aggregates, neither in the frontal cortical tissue nor in the putamen in PSP tissue sections. (Further details are described in the Supplementary Results and Supplementary Figure 4 and 5.)

## Discussion

Using the recently developed tau tracer ^18^F‐AV‐1451, we studied tracer uptake in 11 PSP patients and 11 age‐matched healthy controls and found the highest SUVRs in the basal ganglia in both controls and PSP cases. ROI‐based analyses revealed increased retention of ^18^F‐AV‐1451 in PSP patients in the globus pallidus, putamen, caudate nucleus, thalamus, and midbrain, even when adjusting for age, with the largest effect sizes in the globus pallidus. Furthermore, a cluster corresponding to the globus pallidus was found using a voxelwise approach, although conservative correction for multiple comparisons was not employed because of the limited sample size. This is further corroborated by the positive correlation of the globus pallidus signal to increasing scores on the PSP rating scale, indicating more pronounced pathology with increasing disease severity. Our results are consistent with neuropathologial studies showing that the basal ganglia nuclei, thalamus, and the midbrain are heavily affected by tau pathology early in the disease development of PSP.^16^ However, although the retention of ^18^F‐AV‐1451 is significantly increased in PSP, there is an overlap in PET SUVR values in the globus pallidus between PSP patients and controls, which reduces the clinical use of ^18^F‐AV‐1451 PET as a diagnostic tool in the diagnostic work‐up of PSP. Longitudinal studies in PSP patients are needed to investigate whether ^18^F‐AV‐1451 might be used in clinical trials evaluating new disease modifying medications directed against tau aggregation.

One study has shown increased retention of ^18^F‐AV‐1451 in the basal ganglia in healthy controls^18^ despite the fact that there is no in vitro binding of the ligand in the same structures when using autoradiography.^19^, ^20^ Interestingly, in the present study some of the healthy controls, in addition to all of the PSP patients, exhibited increased retention of ^18^F‐AV‐1451 in the basal ganglia. We also found that this increased retention of ^18^F‐AV‐1451 in the basal ganglia was associated with increasing age in both diagnostic groups. ^18^F‐AV‐1451 has been shown to bind to neuromelanin, for example, in the substantia nigra,^21^ but the reason for the increased retention in the putamen, caudate nucleus, and globus pallidus of healthy controls is still unknown.

Generally the SUVR values were low, with no specific binding seen in cortical areas even in advanced cases of PSP. The low cortical SUVRs and binding potentials *in vivo* were corroborated by an absence of specific binding in vitro using autoradiography of postmortem frontal cortical tissue from PSP patients. The presence of tau aggregates in these PSP tissue samples was confirmed by positive tau immunostaining in adjacent sections. Specific binding of ^3^H‐AV‐1541 to tau aggregates was clearly demonstrated in Alzheimer's disease (AD) brain sections containing abundant tau pathology. The lack of binding observed using autoradiography in tissue from the putamen of a patient with PSP may be a result of the lower amount of tau aggregates observed in PSP sections or to differences in the isoform composition or aggregation ultrastructure of tau in PSP and AD. The type of tau aggregating in the various tau‐related disorders differs when it comes to the type of tau isoforms. The ultrastructure of the inclusions in PSP are straight filaments in tufted astrocytes, coiled bodies, and globose tangle inclusions, mainly consisting of 4R tau, in contrast to the paired helical filament structure of aggregates in neurofibrillary tangles of 3R and 4R tau in AD.^22^, ^23^ The autoradiographic findings of the present study are in line with recently published results showing an absence of^19^, ^20^ or minimal^24^ specific binding of the tracer AV‐1451 to tau aggregates in cortical postmortem samples from PSP cases. The authors suggested that AV‐1451 binds stronger to the paired helical filaments of tau seen in AD compared to the predominantly straight tau filaments typical of PSP.^19^, ^20^, ^24^ The discrepancy between AV‐1451 PET retention in the basal ganglia and the autoradiography could be a result of lower affinity of the radiotracer to tau aggregates *in vitro* than *in vivo*, in addition to an already low binding to straight tau filaments. One argument supporting the notion that the signal in the basal ganglia is indeed derived from binding to tau aggregates is that the globus pallidus, the region with the highest SUVR in this study, is also one of the regions most affected by tau pathology in PSP.^16^

Another possibility could be that AV‐1451, apart from tau aggregates, also binds to another yet unknown structure *in vivo*, which increases with disease severity in the basal ganglia of PSP patients. An argument in favor of this idea is that we see an age‐related increased retention in the basal ganglia also in healthy controls, an area that does not normally contain isolated tau pathology in normal aging.

There are limitations with this study. First, the number of cases in the present study is limited to 11 in each diagnostic group, which might reduce the chance of detecting subtle changes related to tau pathology (statistical type II error). Second, because of the limited number of participants, no correction for multiple comparisons was done. Third, the autoradiography data from the basal ganglia is limited to one case, but results are in line with previously published data.^19^, ^20^

In conclusion, the retention of ^18^F‐AV‐1451 in the basal ganglia is increased in patients with PSP when compared with controls. However, the overlap between the diagnostic groups as well as the age‐dependent increase of the retention in controls makes it difficult to include the method in the diagnostic work‐up of PSP. However, future longitudinal studies are needed to evaluate whether repeated AV‐1451 PET scans can be used to detect changes in the basal ganglia over time in PSP, which could indicate that the method might be used to detect effects of disease‐modifying therapies. Furthermore, we found that the off‐target retention of ^18^F‐AV‐1451 in the basal ganglia increases clearly with age in elderly individuals independent of diagnostic group.

## Authors' Roles

1. Research Project: A. Conception, B. Organization, C. Execution; 2. Statistical Analysis: A. Design, B. Execution, C. Review and Critique; 3. Manuscript Preparation: A. Writing the First Draft, B. Review and Critique.

R.S.: 1A, 1B, 1C, 2A, 2B, 3A

M. Schain: 1C, 2C, 3A

C.N.: 1B, 2C, 3B

O.S.: 1C, 3B

T.O.: 1B, 1C, 2C, 3B

D.H.: 2C, 3B

J.J.: 2C, 3B

E.B.: 2C, 3B

M. Schöll: 2A, 2B, 3B

M.H.: 1B, 1C, 2C, 3A

O.H.: 1A, 1B, 1C, 2A, 3A

## Full financial disclosure for the previous 12 months

R.S., C.N., O.S., T.O., D.H., J.J., and O.H. are employees at Skåne University Hospital, Sweden. R.S. has received funding from Bundy Academy, the Bente Rexed Foundation, and the Swedish federal government under the Governmental funding of clinical research within the National Health Services (ALF) agreement. M. Schain has been an employee of Lund University, Sweden; Karolinska Institutet, Stockholm Sweden; and Columbia University, New York, and has received funding from Karolinska Institute (KI)‐funds for geriatric diseases and the Raymond and Beverly Sackler center. E.B. and M.H. are employees of F. Hoffmann‐La Roche Ltd., Basel, Switzerland. O.H. has received funding stated in the study funding section.

## Supporting information

Additional Supporting Information may be found in the online version of this article at the publisher's web‐site.

Supporting Information 1

Supporting Information Figure 1

Supporting Information Figure 2

Supporting Information Figure 3

Supporting Information Figure 4

Supporting Information Figure 5
