# Supplementary material for: Increased basal ganglia binding of 18 F‐AV‐1451 in patients with progressive supranuclear palsy
Source: Mov Disord. 2016 Oct 6;32(1):108–14. doi: 10.1002/mds.26813 (PMC6204612; doi:10.1002/mds.26813)
Supplement: Supplementary file 1 — Supporting Information 1 [file MDS-32-108-s001.docx]

**Increased basal ganglia binding of ^18^F-AV1451 in patients with PSP**

Ruben Smith, Martin Schain, Christer Nilsson, Olof Strandberg, Tomas Olsson, Douglas Hägerström, Jonas Jögi, Edilio Borroni, Michael Schöll, Michael Honer and Oskar Hansson.

***Supplementary methods***

*Cortical ROIs*

Cortical ROIs derived from the AAL-atlas (within PMOD 3.603) were pooled into:

Frontal (Precentral_l, Precentral_r, Frontal_Sup_l, Frontal_Sup_r, Frontal_Sup_Orb_l, Frontal_Sup_Orb_r, Frontal_Mid_l, Frontal_Mid_r, Frontal_Mid_Orb_l, Frontal_Mid_Orb_r, Frontal_Inf_Oper_l, Frontal_Inf_Oper_r, Frontal_Inf_Tri_l, Frontal_Inf_Tri_r, Frontal_Inf_Orb_l, Frontal_Inf_Orb_r, Supp_Motor_Area_l, Supp_Motor_Area_r, Frontal_Sup_Medial_l, Frontal_Sup_Medial_r, Frontal_Med_Orb_l, Frontal_Med_Orb_r);

Temporal (Temporal_Sup_l, Temporal_Sup_r, Temporal_Pole_Sup_l, Temporal_Pole_Sup_r, Temporal_Mid_l, Temporal_Mid_r, Temporal_Pole_Mid_l, Temporal_Pole_Mid_r, Temporal_Inf_l, Temporal_Inf_r);

Occipital (Calcarine_l, Calcarine_r, Cuneus_l, Cuneus_r, Lingual_l, Lingual_r, Occipital_Sup_l, Occipital_Sup_r, Occipital_Mid_l, Occipital_Mid_r, Occipital_Inf_l, Occipital_Inf_r); and

Parietal areas (Postcentral_l, Postcentral_r, Parietal_Sup_l, Parietal_Sup_r, Parietal_Inf_l, Parietal_Inf_r, Supra_Marginal_l, Supra_Marginal_r, Angular_l, Angular_r, Precuneus_l, Precuneus_r).

Cerebellar grey matter ROIs (Cerebellum_Crus1_l, Cerebellum_Crus1_r, Cerebellum_Crus2_l, Cerebellum_Crus2_r, Cerebellum3_l, Cerebellum3_r, Cerebellum45_l, Cerebellum45_r, Cerebellum6_l, Cerebellum6_r, Cerebellum7_l, Cerebellum7_r, Cerebellum8_l, Cerebellum8_r, Cerebellum9_l, Cerebellum9_r, Cerebellum10_l, Cerebellum10_r) were pooled into one reference region.

*Delineation of subcortical structures*

The caudate and the putamen were delineated based on differences in the signal intensity between the nuclei and the surrounding white matter/globus pallidus in T1-MPRAGE MR images. The ventral boundaries of the nuclei were set as the anterior commisure and the dorsal boundary was defined by the dorsal boundary of putamen. The globus pallidus was delineated between the internal capsule and putamen. Thalamus was delineated using the ventricular borders as well as the intensity differences between the thalamic nuclei and the internal capsule/crus cerebri on T1-MPRAGE images. The dentate nucleus was delineated using a spherical (5mm diameter) ROI in the mediodorsal cerebellar white matter. Similarly, the frontal white matter was defined using a spherical (10mm diameter) ROI located lateral to the dorsolateral boundary of the anterior part of the lateral ventricles. For the delineation of midbrain and pons, a line from the ventral rostral margin of pons to the caudal boundary of tectum was used to demarcate pons from midbrain. The posterior boundary of pons was set as the 4th ventricle. The caudal boundary was defined as a perpendicular line through the brain stem from the medullopontine sulcus. The midbrain was delineated rostrally by the thalamus. Volumetric estimates were obtained from the ROI volumes in MR space. The manual delineation of all ROIs was made in a random subject order, by a single rater, blinded to both PET data and diagnostic group.

*Radiolabeling of ^18^F-AV1451*

^18^F-Fluoride was generated using a GE PETtrace cyclotron at Skåne University Hospital. ^18^F-Fluoride activity was retained on a pre-conditioned Sep-Pak® Light Accell™ Plus (QMA) Cartridge and eluted using 0.75 mL of a 0.075 M Tetrabutylammonium Hydrogen Carbonate Solution. The eluted activity was heated to 110 °C under nitrogen flow and vacuum for 5min 20s while acetonitrile was added three times to facilitate the azeotropic removal of water. AV-1622 [1.5 mg in anhydrous DMSO (1.6 mL)] was added and the resulting mixture was kept at 120 °C for 5 min followed by de-protection using 1.0 mL of 2M HCl(aq) at 100 °C for 5 min. After cooling for 1min, the crude ^18^F-AV1451 mixture was neutralized with 2.4 ml of 1M NaOH(aq). The resulting mixture was passed through an Oasis® HLB Light cartridge. The retained ^18^F-AV1451 was washed with water for injection (WFI) before being eluted off the Oasis HLB Light cartridge using 1.5 mL of acetonitrile. The crude ^18^F-AV1451 was diluted with 3.5 ml of WFI/Ethanol (10/1) and loaded onto a semi-preparative Zorbax Eclipse XDB-C18, 9.4 x 250 mm, 5μm HPLC column for purification using the isocratic elution 40 % ethanol /60 % 100 mM phosphate buffer at a 4 mL/min flow rate. The HPLC fraction containing the purified ^18^F-AV1451 was collected (4 mL) and directly transferred into the final vial through a 0.22 µm Cathivex-GS filter. Radiochemical purity was 99.56 ± 0.08 %. The pH of the solution was adjusted with 2 ml of citrate buffer and the solution was diluted with 10 mL NaCl 0.9 %.

*In vitro autoradiography*

Fresh frozen frontal cortex tissue blocks from 3 PSP patients and 3 healthy controls were obtained from the Banner Sun Health Research Institute (Sun City, AZ, USA). A mid-temporal gyrus block with high tau load from a late stage Alzheimer’s disease patient was also purchased from the Banner Sun Health Research Institute. A tissue block from putamen from a patient with PSP was kindly provided by Dr. John van Swieten, Dutch Brain Bank. Ten µm thick sections of the brain tissue blocks were generated with a cryostat (Leica CM3050) at -17 °C chamber temperature and -15 °C object temperature. Sections were transferred to Histobond+ microscope slides (Marienfeld Laboratory Glasware). After drying for 3 hours at room temperature the sections were stored at -20 °C.

^3^H-AV1451 was tritiated at Roche with a specific activity of 29 Ci/mmol and a radiochemical purity higher than 99%. The brain sections from PSP patients, healthy control and Alzheimer’s disease patients were incubated with the radioligand (3 nM (Figure 2) or 30 nM (Suppl figure 1)) in 50 mM Tris buffer, pH 7.4 at room temperature for 30 min. After washing 3x 10 min at 4 °C in 50 mM Tris buffer, pH 7.4 and 3 quick dips in H_2_O dist. at 4 °C, the sections were dried at 4°C for 3 h. The sections were placed in a FujiFilm Cassette (BAS 2025), exposed with a FujiFilm Imaging Plates (BAS-IP TR 2025) for 5 days and afterwards scanned with a FujiFilm IP reader (BAS-5000) with a resolution of 25 µm per pixel. The autoradiograms were visualized with the software MCID analysis (version 7.0, Imaging Research Inc.). Non-specific binding of ^3^H-AV1451 was assessed by co-incubation with 10 µM cold AV1451. The experimental protocol was set up and optimized to give a robust signal from positive controls.

The presence of tau aggregates was confirmed on adjacent sections using the tau-specific antibody pS422-conjugated with Alexa555 (5 µg/mL)^1^.

*Voxel-based Morphometry*

Imaging was performed on a 3.0T Siemens Skyra scanner (Siemens Medical Solutions, Erlangen, Germany). The high-resolution 3D T1-weighted volume used for Voxel Based Morphometry (VBM) was acquired using an MPRAGE sequence (in-plane resolution=1×1mm^2^, slice thickness=1mm, TR=1900ms, TE=2.54ms, flip-angle=9°).

Structural data was analysed with FSL-VBM^2^ (<http://fsl.fmrib.ox.ac.uk/fsl/fslwiki/FSLVBM>), an optimised VBM protocol^3^ carried out with FSL tools^4^. First, structural images were brain-extracted and grey matter-segmented before being registered to the MNI152 standard space using non-linear registration. The resulting images were averaged and flipped along the x-axis to create a left-right symmetric, study-specific grey matter template. Second, all native grey matter images were non-linearly registered to this study-specific template and modulated to correct for local expansion (or contraction) due to the non-linear component of the spatial transformation. The modulated grey matter images were then smoothed with an isotropic Gaussian kernel with a sigma of 4 mm. Finally, voxelwise GLM was applied using permutation-based non-parametric testing, correcting for multiple comparisons across space using a cluster based method with p=0.01.

***Supplementary results***

*Autoradiography*

Immunohistochemistry, using a tau specific antibody that recognizes an epitope that includes phosphorylated serine 422, revealed the presence of globose tau tangles in all three cortical PSP tissue blocks and in the tissue from the putamen, while the three healthy control tissue blocks were devoid of any pS422 antibody staining (Suppl. figure 3). Notably, the density of tau aggregate staining in PSP tissue sections was much lower compared to late stage AD cortical tissue (Suppl. Figure 4). We found no specific binding of ^3^H-AV1451 to tau aggregates, neither in frontal cortical tissue, nor in the putamen in PSP tissue sections. Binding intensities in autoradiograms were indistinguishable in PSP and healthy control tissue sections (Suppl. Figure 4, A and B vs. C, D and E) and radioligand binding in PSP tissue sections could not be blocked by co-incubation with unlabeled AV1451 (Suppl. figure 3). On the contrary, specific binding of ^3^H-AV1451 to tau aggregates in a late stage AD cortex section was clearly demonstrated on the macroscopic and the microscopic level (Suppl. Figure 4F and H), which could be largely blocked by a higher concentration of cold AV1451 (Suppl. figure 3).

**Supplementary Table 1:**

| **Study-ID** | **Clinical diagnosis** | **Amyloid status** | **MMSE** | **Temporal lobe SUVR** | **Globus Pallidus SUVR** |
| --- | --- | --- | --- | --- | --- |
| PSP 1 | RS | Normal | 30 | 1.03 | 1.69 |
| PSP 2 | RS | Normal | 22 | 1.05 | 2.10 |
| PSP 3 | RS | Normal | 28 | 1.05 | 1.58 |
| PSP 4 | RS | Normal | 26 | 1.02 | 1.84 |
| PSP 5 | RS | Normal | N/A | 1.06 | 1.73 |
| PSP 6 | RS | Normal | N/A | 1.05 | 1.75 |
| PSP 7 | RS | Abnormal | 29 | 1.08 | 1.49 |
| PSP 8 | PSP-P | Normal | 29 | 1.04 | 1.76 |
| PSP 9 | PSP-P | Normal | 24 | 1.08 | 1.99 |
| PSP 10 | RS | N/A | 28 | 1.13 | 1.67 |
| PSP 11 | RS | Abnormal | 29 | 1.13 | 1.71 |
| Control 1 | - | Normal | 27 | 1.12 | 1.39 |
| Control 2 | - | Normal | 28 | 0.99 | 1.24 |
| Control 3 | - | Normal | 30 | 1.16 | 1.35 |
| Control 4 | - | Abnormal | 30 | 1.14 | 1.40 |
| Control 5 | - | Normal | 30 | 1.08 | 1.50 |
| Control 6 | - | Normal | 29 | 1.02 | 1.64 |
| Control 7 | - | Normal | 28 | 1.08 | 1.84 |
| Control 8 | - | Normal | 30 | 1.14 | 1.83 |
| Control 9 | - | Normal | 30 | 1.10 | 1.62 |
| Control 10 | - | Normal | 30 | 1.17 | 1.50 |
| Control 11 | - | Abnormal | 30 | 1.09 | 1.24 |

RS - Richardson's syndrome; PSP-P - PSP-Parkinsonism. Amyloid status was determined by CSF β-amyloid (cut off 500 ng/l) or by ^18^F-Flutemetamol (composite score SUVR cut off 1.51) (for methodological reference see^5^). Mann-Whitney U-test revealed no significant differences between patients with normal or abnormal amyloid status in any of the analysed regions including temporal lobe and globus pallidus. N/A - Not available. Two patients in the PSP group could not be tested with MMSE due to mutism. The patients with abnormal amyloid showed no signs of increased ^18^F-AV-1451 retention in the temporal lobes, and the distribution of ^18^F-Flutemetamol was clearly different from the pattern seen using ^18^F-AV-1451.

***Supplementary references***

1. Grueninger F, Bohrmann B, Czech C, et al. Phosphorylation of Tau at S422 is enhanced by Abeta in TauPS2APP triple transgenic mice. Neurobiol Dis 2010;37(2):294-306.

2. Douaud G, Smith S, Jenkinson M, et al. Anatomically related grey and white matter abnormalities in adolescent-onset schizophrenia. Brain 2007;130(Pt 9):2375-2386.

3. Good CD, Johnsrude IS, Ashburner J, Henson RN, Friston KJ, Frackowiak RS. A voxel-based morphometric study of ageing in 465 normal adult human brains. Neuroimage 2001;14(1 Pt 1):21-36.

4. Smith SM, Jenkinson M, Woolrich MW, et al. Advances in functional and structural MR image analysis and implementation as FSL. Neuroimage 2004;23 Suppl 1:S208-219.

5. Palmqvist S, Zetterberg H, Mattsson N, et al. Detailed comparison of amyloid PET and CSF biomarkers for identifying early Alzheimer disease. Neurology 2015;85(14):1240-1249.
