# Supplementary figures and images for: Increased basal ganglia binding of 18 F‐AV‐1451 in patients with progressive supranuclear palsy
Source: Mov Disord. 2016 Oct 6;32(1):108–14. doi: 10.1002/mds.26813 (PMC6204612; doi:10.1002/mds.26813)

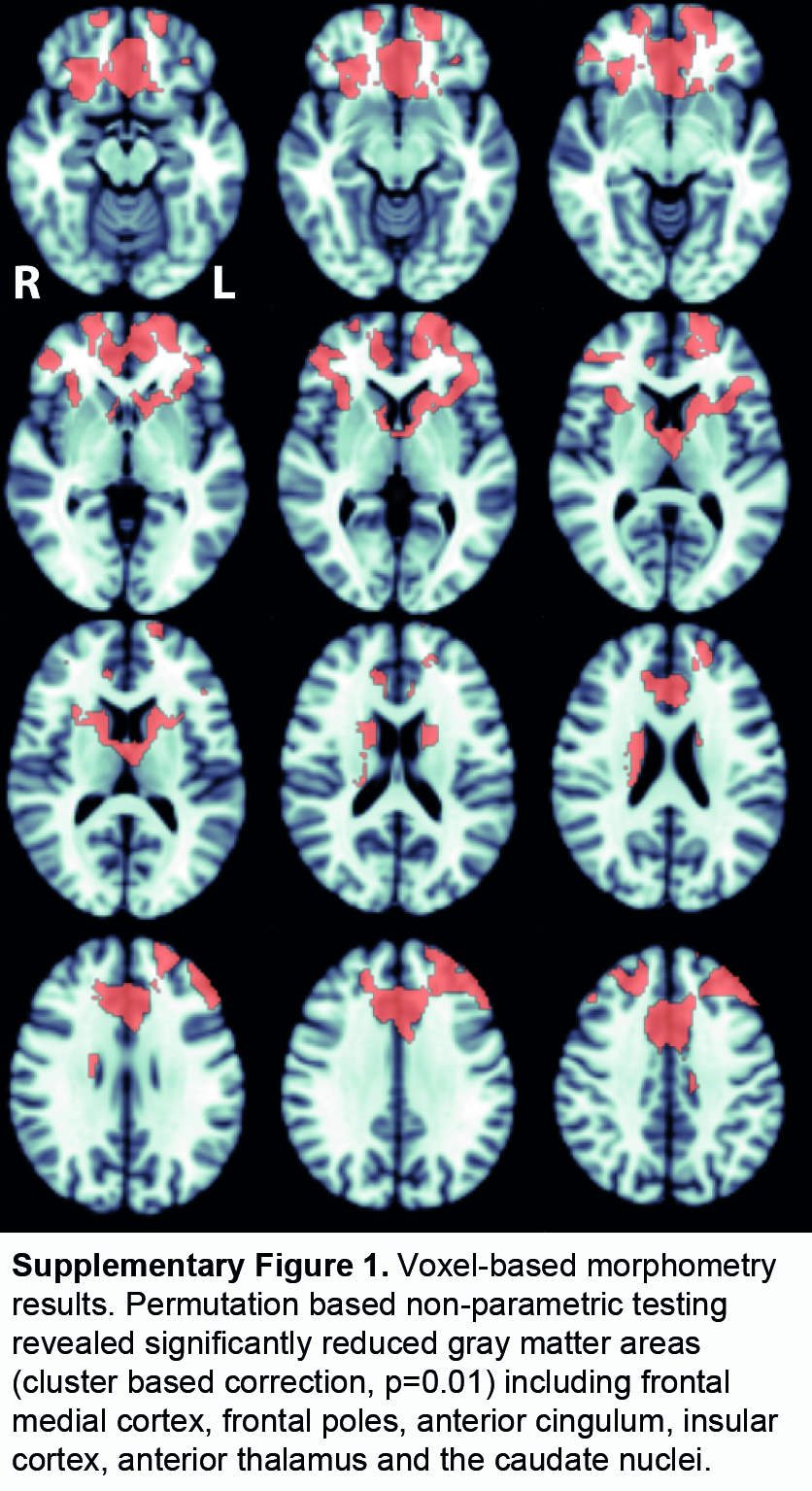

Supplement: Supplementary file 2 — Supporting Information Figure 1 [file MDS-32-108-s002.tif]

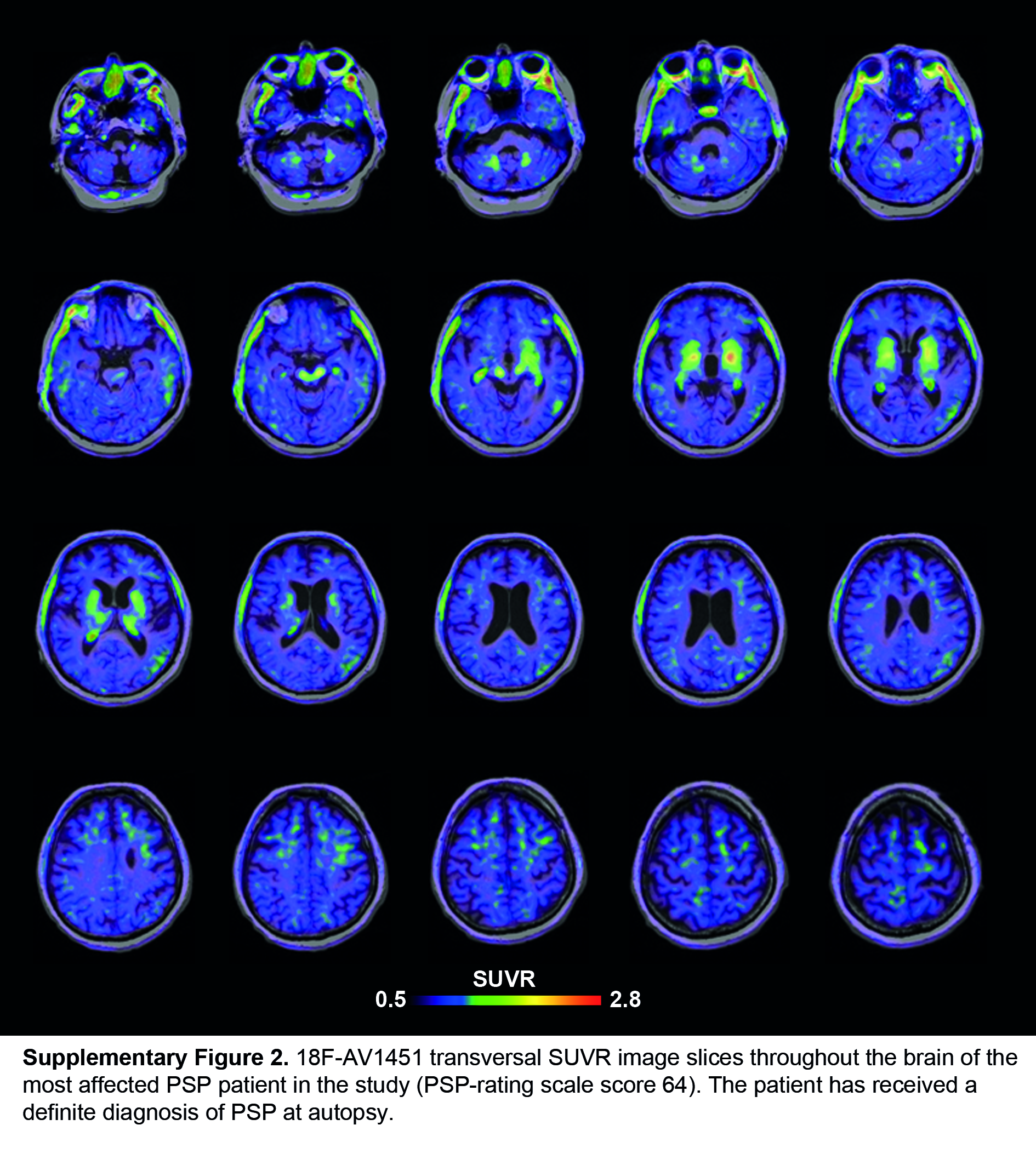

Supplement: Supplementary file 3 — Supporting Information Figure 2 [file MDS-32-108-s003.tif]

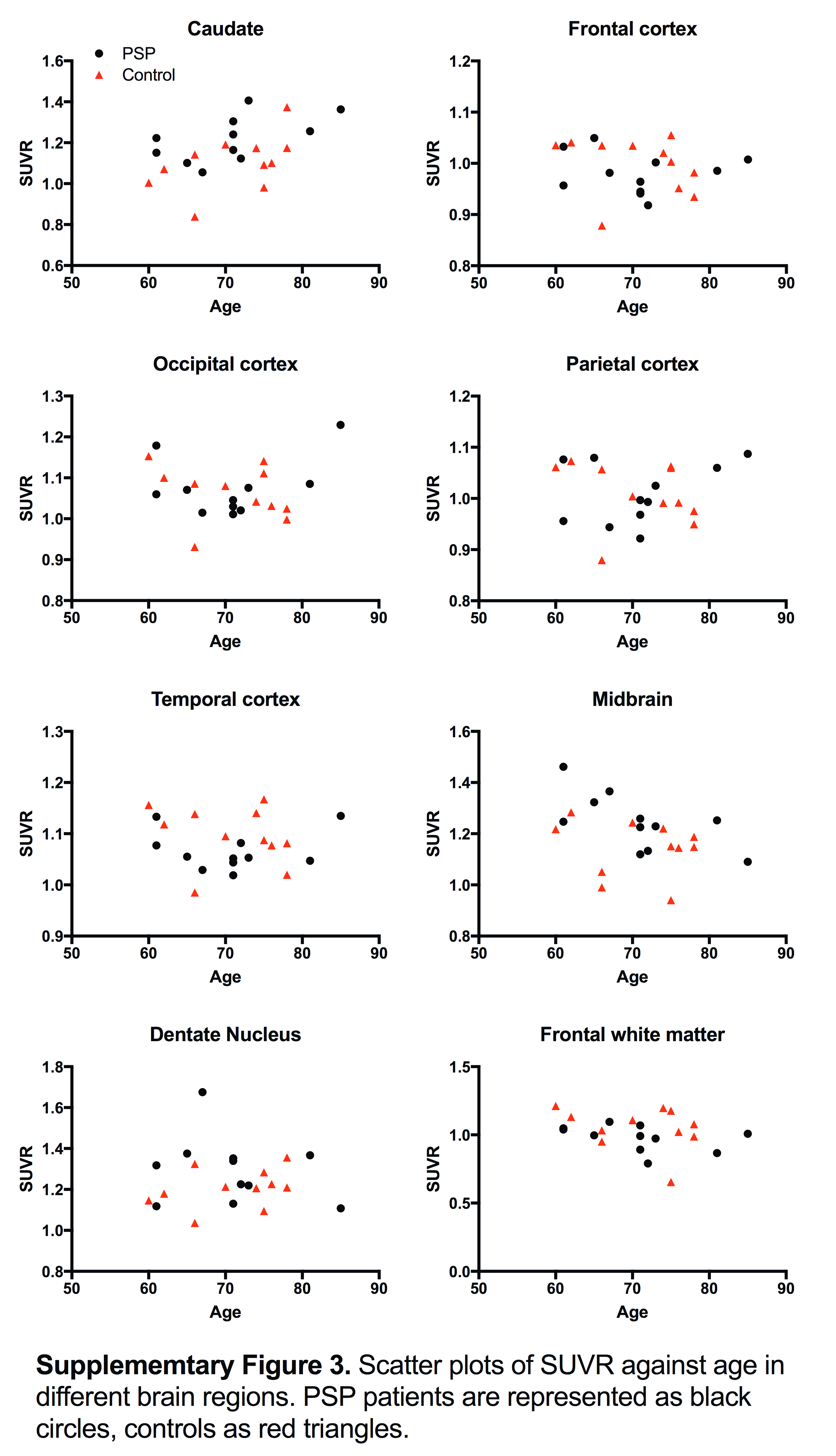

Supplement: Supplementary file 4 — Supporting Information Figure 3 [file MDS-32-108-s004.tiff]

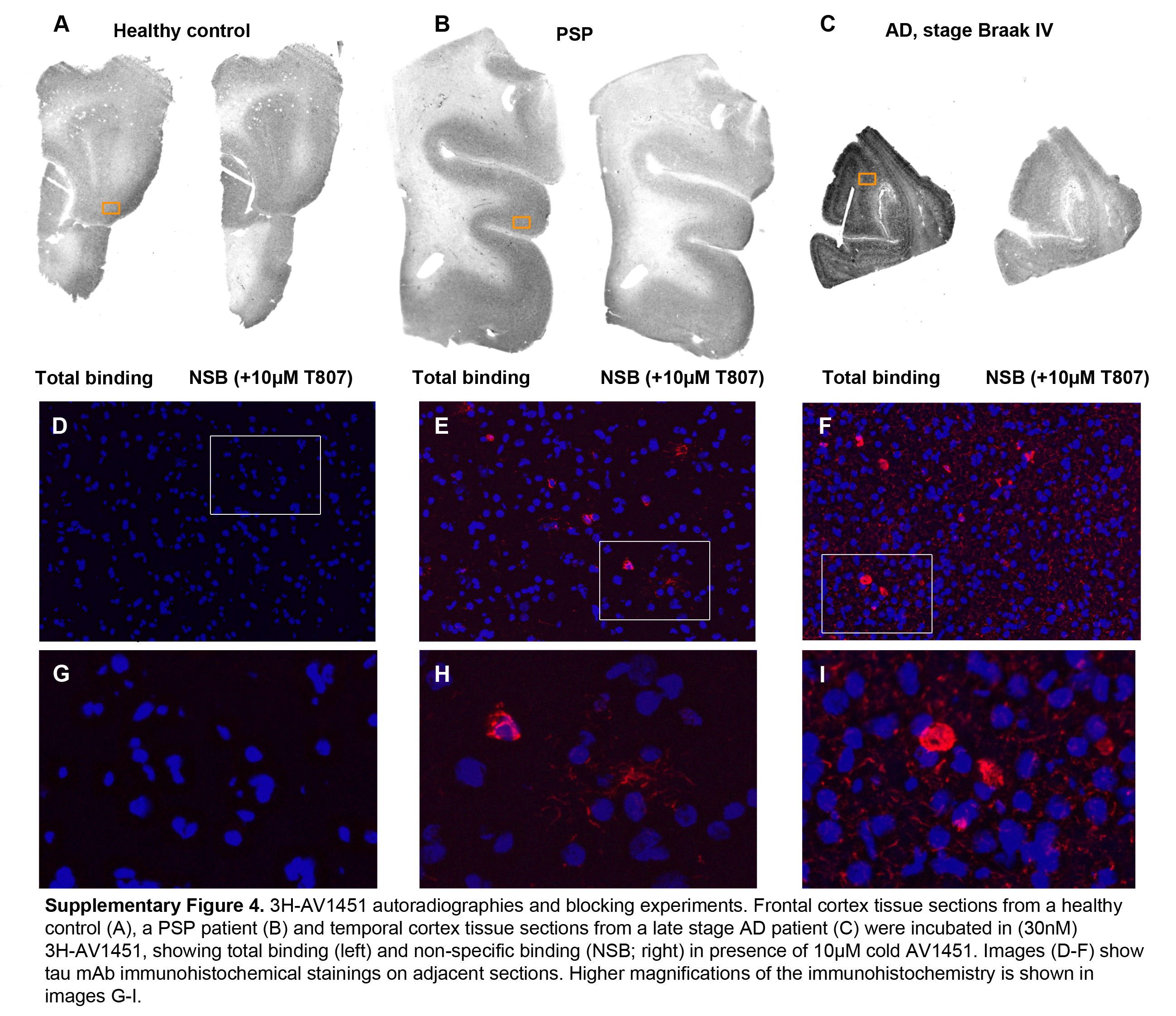

Supplement: Supplementary file 5 — Supporting Information Figure 4 [file MDS-32-108-s005.tif]

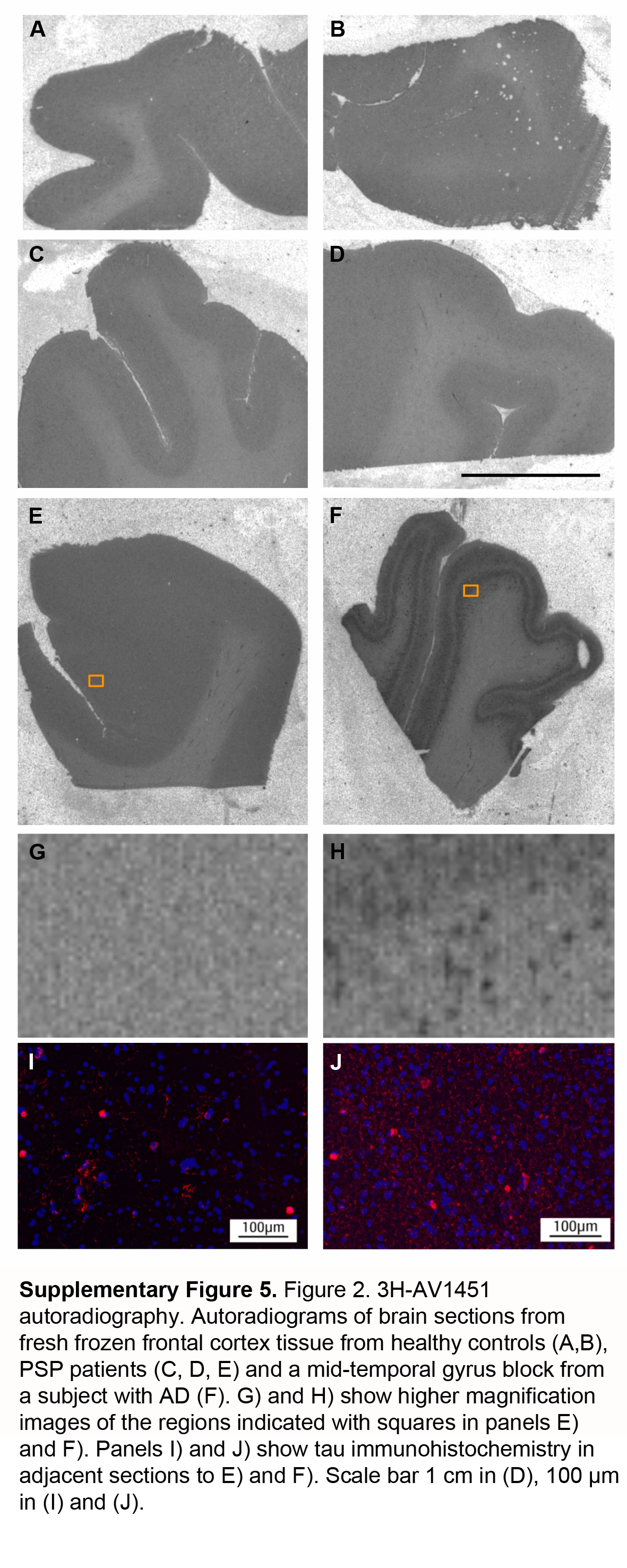

Supplement: Supplementary file 6 — Supporting Information Figure 5 [file MDS-32-108-s006.tif]
